# Perioperative stellate ganglion block improves early postoperative sleep after gynecological laparoscopic surgery: a randomized controlled trial

**DOI:** 10.3389/fnins.2026.1820566

**Published:** 2026-04-10

**Authors:** Qian Yang, Xuelian Yang, Tong Mu, Wei Chen

**Affiliations:** Department of Anesthesiology, The First Affiliated Hospital of Chongqing Medical University, Chongqing, China

**Keywords:** autonomic modulation, laparoscopic surgery, postoperative recovery, postoperative sleep disturbance, stellate ganglion block

## Abstract

**Background:**

Postoperative sleep disturbance is highly prevalent after laparoscopic surgery and is closely associated with perioperative stress responses, impaired recovery quality, and prolonged hospital stay. However, effective interventions specifically targeting postoperative sleep remain limited. This study aimed to evaluate the effect of perioperative stellate ganglion block (SGB) on postoperative sleep in patients undergoing gynecological laparoscopic surgery.

**Methods:**

This single-center, prospective, randomized controlled trial enrolled eligible patients undergoing elective gynecological laparoscopic surgery, who were randomly assigned to the SGB group or the control group. Patients in the SGB group received an ultrasound-guided right stellate ganglion block before anesthesia induction. Postoperative sleep quality on postoperative days 1 and 2 was assessed using the Athens Insomnia Scale (AIS) and self-reported Total Sleep Time (TST) as the primary outcomes. Secondary outcomes included postoperative nausea and vomiting (PONV), analgesia-related variables, perioperative hemodynamic changes, and length of hospital stay.

**Results:**

Compared with the control group, patients in the SGB group had significantly lower AIS scores and longer TST on postoperative days 1 and 2. The incidence of postoperative sleep disturbance was reduced, and hospital length of stay was significantly shorter in the SGB group. No significant differences were observed in preoperative sleep status between the two groups.

**Conclusion:**

Perioperative stellate ganglion block significantly improves early postoperative sleep in patients undergoing gynecological laparoscopic surgery. Improvement in sleep may represent an important pathway through which SGB facilitates perioperative recovery.

**Clinical trial registration:**

https://www.chictr.org.cn/showproj.html?proj=212749, identifier (ChiCTR2300078051).

## Introduction

Postoperative sleep disturbances are a common yet often underestimated issue in the perioperative period, occurring in 15 to 72% of surgical patients, with a higher prevalence observed in female patients (Fang et al., 2025; Qiu et al., 2022). These disturbances are associated with multiple factors, including postoperative pain and anxiety, postoperative nausea and vomiting, and poor adaptation to the hospital environment (Du et al., 2025; Butris et al., 2023). Postoperative sleep disturbances may contribute to postoperative delirium and cognitive dysfunction, exacerbate acute postoperative pain, and delay recovery (Yu et al., 2022). Therefore, improving postoperative sleep has increasingly been recognized as an important component of optimizing perioperative recovery. Currently, interventions targeting postoperative sleep remain limited. Conventional analgesic or sedative strategies can partially improve sleep but are often accompanied by adverse effects such as respiratory depression, delirium, or delayed recovery, which restrict their perioperative application (Soh et al., 2024). However, to our knowledge, there are currently no studies investigating preventive interventions for postoperative sleep disturbances (PSD). Therefore, exploring an intervention that can improve sleep without introducing additional risks holds significant clinical relevance.

The stellate ganglion, as a key node of the cervical sympathetic chain, plays a critical role in regulating autonomic balance, stress responses, and circadian rhythms (Tsai et al., 2026; Shi et al., 2023). Stellate ganglion block (SGB) has been applied in various conditions associated with sympathetic overactivity and has shown potential effects in modulating sleep and mood (Zhang et al., 2024; Yang et al., 2023). Although SGB appears to be safe and potentially effective, further prospective clinical studies are still needed to definitively confirm its efficacy in the perioperative setting.

Therefore, this study, using postoperative sleep as the primary outcome and a randomized controlled design, aimed to investigate the effect of perioperative SGB on postoperative sleep quality in patients undergoing gynecological laparoscopic surgery and to further explore its relationship with perioperative recovery-related outcomes.

## Methods

### Study design and ethical approval

This study was designed as a prospective, single-center, randomized, double-blind controlled trial and was conducted at the First Affiliated Hospital of Chongqing Medical University. The study protocol was approved by the institutional ethics committee (approval No. 2023–108) and was registered on a clinical trial registry prior to study initiation (registration No. ChiCTR2300078051). Written informed consent was obtained from all participants before enrollment. The study was conducted in accordance with the principles of the Declaration of Helsinki.

### Inclusion criteria

Women aged ≥18 years scheduled for gynecologic laparoscopic surgery under general anesthesia with an estimated duration exceeding 1 h were eligible for enrollment, and American Society of Anesthesiologists (ASA) physical status classification of I–III, and New York Heart Association (NYHA) functional class I–II, with no clinically significant dysfunction of major organ systems. Willingness to receive preoperative stellate ganglion block and to use patient-controlled intravenous analgesia (PCIA) postoperatively, with agreement to participate in postoperative follow-up.

### Exclusion criteria

1. Contraindications to needle puncture, including coagulation disorders, allergy to local anesthetics, cervical skin infection, or systemic infection;
2. Presence of diabetes mellitus or severe cardiac, cerebrovascular, renal, or other major systemic diseases;
3. Cognitive impairment, psychiatric disorders, or inability to adequately understand or cooperate with postoperative assessments;
4. Conversion from laparoscopic surgery to open surgery during the procedure.

### Randomization and blinding

This study was conducted as a prospective, randomized, assessor-blinded controlled trial. Patients were randomly assigned in a 1:1 ratio to the stellate ganglion block (SGB) group or the control group using a computer-generated randomization sequence. Group allocation was concealed in opaque, sealed envelopes prepared and maintained by an independent investigator. The anesthesiologist performing the stellate ganglion block did not participate in intraoperative management or postoperative follow-up. Postoperative outcome assessment and statistical analyses were conducted by investigators who were blinded to group allocation.

### Anesthesia and perioperative management

All patients received a standardized general anesthesia protocol. Upon arrival in the operating room, routine monitoring was established, including electrocardiography, noninvasive blood pressure, pulse oxygen saturation, and end-tidal carbon dioxide concentration. Anesthesia was induced with midazolam (0.05–0.1 mg/kg), propofol (1.5–2.5 mg/kg), sufentanil (0.25–0.6 μg/kg), and vecuronium (0.1–0.15 mg/kg). After tracheal intubation, anesthesia was maintained with anesthetic agents adjusted according to clinical requirements. Intraoperative analgesia, neuromuscular blockade management, and fluid therapy were administered according to a unified protocol. Postoperatively, all patients received the same multimodal analgesic regimen, including patient-controlled intravenous analgesia (PCA), to minimize the potential influence of analgesic differences on study outcomes.

### Stellate ganglion block procedure

An experienced anesthesiologist performed the stellate ganglion block under sterile conditions using real-time ultrasound guidance. Patients in the SGB group underwent ultrasound-guided right stellate ganglion block before induction of anesthesia. The procedure was performed using an ultrasound system (Navii, Wisonic, China).

**Figure 1:**
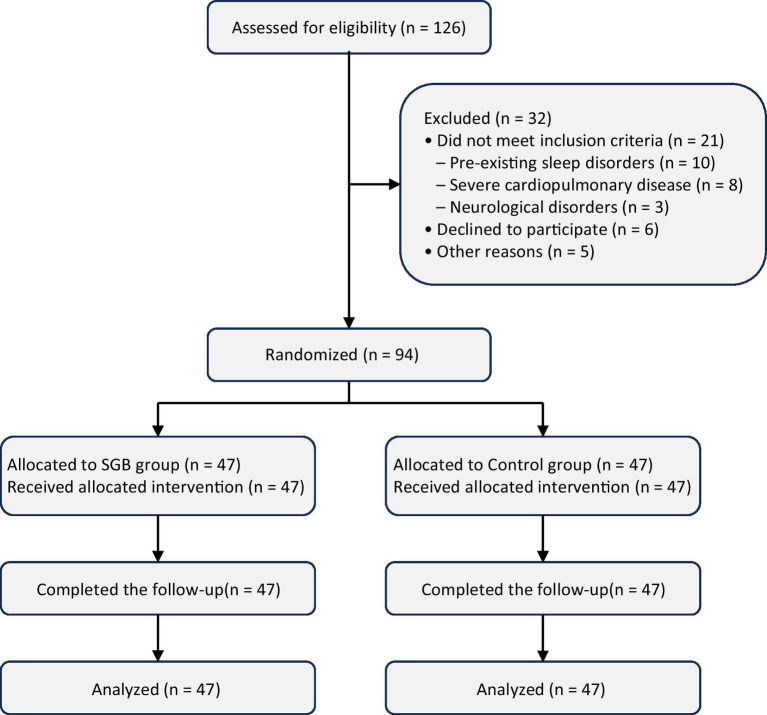
Anatomical structures and needle trajectory for ultrasound-guided right stellate ganglion block. The yellow arrow indicates the prevertebral fascia, and the red asterisk denotes the target site for local anesthetic injection. SCM, sternocleidomastoid muscle; IJV, internal jugular vein; CCA, common carotid artery; LCM, longus colli muscle.

Patients were placed in the supine position with the head slightly extended and rotated to the contralateral side. A high-frequency linear transducer (5–13 MHz) was positioned transversely at the level of the sixth cervical vertebra. Relevant anatomical structures, including the common carotid artery, internal jugular vein, prevertebral fascia, and longus colli muscle, were identified. A nerve block needle (21G, HAKKO, Japan) was advanced under ultrasound guidance, and the needle tip was positioned anterior to the longus colli muscle and beneath the prevertebral fascia. After negative aspiration for blood, 5 mL of 0.25% ropivacaine was slowly injected (Figure 1). Successful blockade was clinically confirmed by the presence of ipsilateral Horner syndrome–related signs, such as ptosis, miosis, or increased facial temperature. Patients in the control group underwent a blinded sham procedure consisting of ultrasound scanning only, without needle insertion or local anesthetic injection.

### Martphone screen time

Sleep-related behavioral data were collected using the HUAWEI Health® application (iOS® and Android®) in conjunction with a HUAWEI® Band GT2 wearable device (HUAWEI Device Co., Ltd., Shenzhen, China). Participants were instructed to wear the device continuously, including during sleep, on either wrist. The device was positioned approximately one finger-width proximal to the wrist bone and secured with a snug fit to ensure direct contact between the sensor and the skin (Zhang et al., 2023; Say et al., 2025).

### Outcome measures

The primary outcome was postoperative sleep quality. Sleep status was assessed using the Athens Insomnia Scale (AIS) preoperatively (D0) and on postoperative day 1 (D1) and day 2 (D2). AIS scores of 4–6 were defined as suspected sleep disturbance, and scores greater than 6 were defined as insomnia. TST was concurrently recorded.

Secondary outcomes included:

1. Incidence of postoperative nausea and vomiting (PONV) on postoperative days 1 and 2;
2. Early postoperative comfort, assessed using the Brüggrman Comfort Scale (BCS) at 30 min, 6 h, 12 h, and 24 h after surgery;
3. Postoperative pain, including headache, shoulder pain, and sore throat, assessed using the visual analog scale (VAS);
4. Perioperative hemodynamic variables, including heart rate (HR) and mean arterial pressure (MAP), recorded before anesthesia induction, at skin incision, 30 min after pneumoperitoneum, and at tracheal extubation;
5. Analgesia-related variables during the perioperative period, including patient-controlled analgesia (PCA) use;
6. Postoperative recovery-related outcomes, including time to first flatus and length of hospital stay.

### Data collection

Based on previous studies, the incidence of postoperative sleep disturbance on postoperative day 1 ranges from 15 to 72% (Zhou et al., 2025). Sample size estimation was performed using PASS 15.0 software. According to preliminary data, the proportion of patients with AIS scores <4 on postoperative day 1 was 55% in the SGB group and 23% in the control group. Sleep disturbance was defined as an AIS score ≥4. With a two-sided *α* of 0.05 and 80% power, 76 participants were required. Allowing for a 20% dropout rate, the total sample size was adjusted to 94 and rounded up to 94 participants (47 per group).

### Statistical analysis

Statistical analyses were performed according to the intention-to-treat (ITT) principle using SPSS software (version 21.0). Continuous variables were tested for normality. Data with a normal distribution are presented as mean ± standard deviation (SD) and were compared using the independent-samples t test. Non-normally distributed data are presented as median (interquartile range, IQR) and were analyzed using the Mann–Whitney U test. Categorical variables are expressed as number (percentage) and were compared using the chi-square test or Fisher’s exact test, as appropriate. For the primary outcome and key secondary outcomes, Bonferroni correction was applied to account for multiple comparisons, and adjusted significance levels were used. Other outcomes were considered exploratory and were not adjusted for multiple comparisons. A two-sided *p* value < 0.05 was considered statistically significant.

## Results

### Study population and baseline characteristics

**Figure 2:**
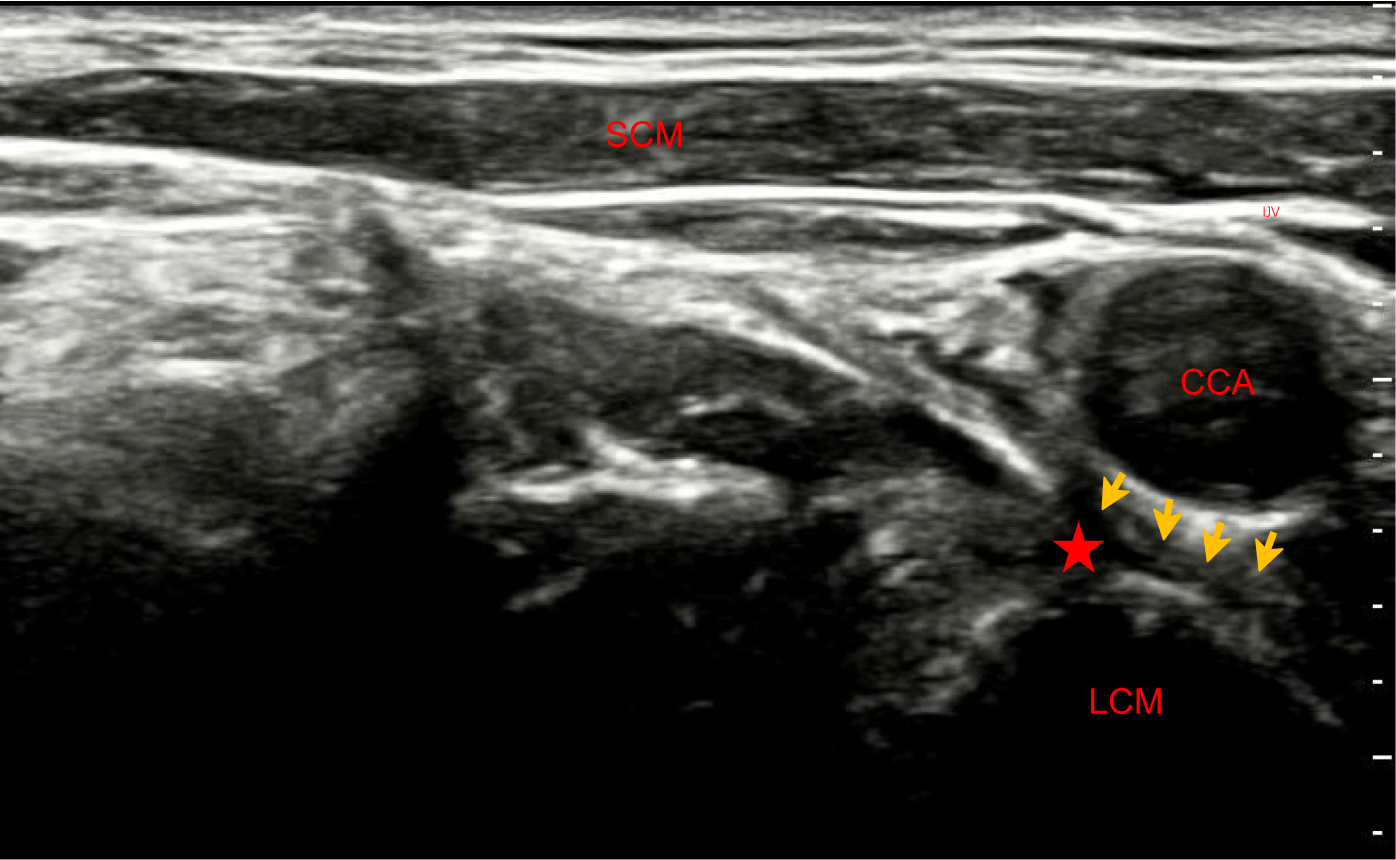
Flow diagram of patient enrollment, randomization, follow-up, and analysis. The diagram illustrates the process of patient screening, random allocation, follow-up, and inclusion in the final analysis.

A total of 126 patients were assessed for eligibility, of whom 32 were excluded before randomization. The remaining 94 patients were randomly assigned to the SGB group or the control group, with 47 patients in each group. All randomized patients completed follow-up and were included in the final analysis (Figure 2).

There were no significant differences between the two groups with respect to baseline characteristics, including age, smoking history, history of nausea and vomiting, comorbidities, preoperative TST, preoperative Athens Insomnia Scale (AIS) scores, and preoperative comfort scores (all *p* > 0.05), indicating good comparability between groups (Table 1). In addition, no significant differences were observed between groups in surgery-related characteristics, including anesthesia duration, pneumoperitoneum duration, opioid consumption, intraoperative blood loss, and fluid administration (Table 2).

**Table 1:** Baseline demographic and preoperative characteristics of patients in the two groups.

Item	Control group (*n* = 47)	SGB group (*n* = 47)	*p* value
Age (years)	54.36 ± 10.17	55.13 ± 8.69	0.493
Non-smoker, n(%)	43 (91.5%)	43 (91.5%)	1.000
No history of nausea and vomiting, n (%)	44 (93.6%)	44 (93.7%)	1.000
No comorbidities, n (%)	40 (85.1%)	40 (85.2%)	1.000
TST D0 (h)	7.00 (1.00)	6.5 (1.00)	0.737
AIS D0 n(%)			0.900
<4	26 (55.3%)	25 (53.2%)	
4–6	17 (36.2%)	20 (42.5%)	
>6	4 (8.5%)	2 (4.3%)	
BCS D0	4 (0)	4 (0)	1.000
Preoperative pain, n (%)			
Head	8 (17.0%)	9 (19.1%)	0.897
Shoulders	4 (8.5%)	3 (6.4%)	0.678
Viscera	8 (17.0%)	7 (14.9%)	0.789

**Table 2:** Perioperative surgical and anesthesia-related characteristics of patients in the two groups.

Item	Control group (*n* = 47)	SGB group (*n* = 47)	*p* value
Anesthesia (h)	4 (0.90)	3.8 (0.95)	0.236
Pneumoperitoneum (h)	2.7 (0.95)	2.5 (1.00)	0.19
Total dose of sufentanil (μg)	40 (0)	40 (0)	0.43
Total fluid infusion (mL)	1,100 (0)	1,100 (500)	0.038
Total hemorrhage (mL)	80 (50)	50 (50)	0.24

Data are presented as mean ± standard deviation or median (interquartile range). SGB, stellate ganglion block.

Data are presented as mean ± standard deviation, median (interquartile range), or number (percentage). SGB, stellate ganglion block; AIS, Athens Insomnia Scale; BCS, Brüggrman Comfort Scale; D0, preoperative period.

### Primary outcome: postoperative sleep quality

**Figure 3:**
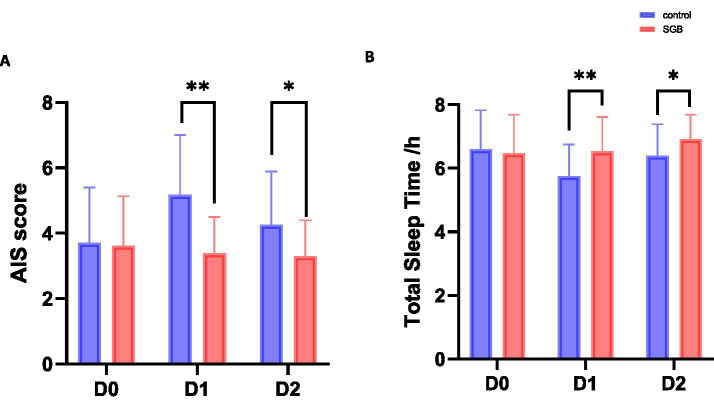
Effects of stellate ganglion block on postoperative sleep quality. **(A)** Changes in Athens Insomnia Scale (AIS) scores at baseline (D0) and on postoperative days 1 (D1) and 2 (D2). **(B)** Changes in total total sleep time at different time points in the two groups. Data are presented as mean ± standard deviation. *p < 0.05, **p < 0.01 compared with the control group.

Preoperative (D0) sleep status was comparable between the two groups. There was no significant difference in the distribution of Athens Insomnia Scale (AIS) scores (*p* = 0.90), and TST was also similar (6.60 ± 1.23 h vs. 6.47 ± 1.22 h, *p* = 0.614). In the early postoperative period, sleep patterns changed in both groups, with more pronounced improvement observed in the SGB group. In terms of AIS score distribution, a significant between-group difference was observed on postoperative day 1 (D1; *p* < 0.001). The proportion of patients with AIS < 4 was markedly higher in the SGB group compared with the control group (59.6% vs. 21.3%), and no patients with AIS > 6 were observed in the SGB group (0% vs. 19.1%). This difference persisted on postoperative day 2 (D2; *p* = 0.008), characterized by a higher proportion of low AIS scores and a lower proportion of high scores in the SGB group. Analysis of total AIS scores showed that the SGB group had significantly lower scores than the control group on both D1 and D2 (D1: 3.38 ± 1.18 vs. 5.17 ± 1.81, *p* = 0.002; D2: 3.30 ± 1.09 vs. 4.25 ± 1.62, *p* = 0.034). Regarding TST, patients in the SGB group had significantly longer sleep time on D1 compared with the control group (6.53 ± 1.09 h vs. 5.76 ± 0.99 h, *p* < 0.002), and this difference remained on D2 (6.91 ± 0.77 h vs. 6.39 ± 0.99 h, *p* = 0.012; Figure 3; Table 3; Supplementary Table S1). Considering the potential influence of menopause on sleep in female patients, a stratified analysis was performed using 51 years as the median menopausal age based on previous literature (Baker et al., 2018). No significant difference in preoperative AIS scores was observed between groups (*p* > 0.05). On postoperative days 1 and 2, AIS scores in the SGB group were significantly lower than those in the control group across age strata (all *p* < 0.05). Interaction analysis showed no significant interaction between age and treatment effect (*P* for interaction > 0.05), suggesting that the effect of SGB on postoperative sleep was consistent across different age groups (Supplementary Tables S1, S2). Overall, these findings indicate that SGB is associated with improved postoperative sleep quality and may contribute to enhanced postoperative recovery.

**Table 3:** Comparison of pre- and postoperative AIS scores and TST between groups.

Item	control group (*n* = 47)	SGB group (*n* = 47)	*p* value
AIS D1(points)	5.17 ± 1.81	3.38 ± 1.18	0.002*
AIS D2(points)	4.25 ± 1.62	3.3 ± 1.09	0.034*
TST(h)			
D0	6.60 ± 1.23	6.47 ± 1.22	0.614
D1	5.76 ± 0.99	6.53 ± 1.09	<0.002
D2	6.39 ± 0.99	6.91 ± 0.77	0.012

Data are presented as mean ± standard deviation (SD). AIS, Athens Insomnia Scale. AIS: Athens Insomnia Scale; D0: preoperative; D1: postoperative day 1; D2: postoperative day 2. *p* values represent between-group comparisons and are adjusted for multiple comparisons. *indicates statistical significance.

### Secondary outcomes

#### Recovery outcomes

**Figure 4:**
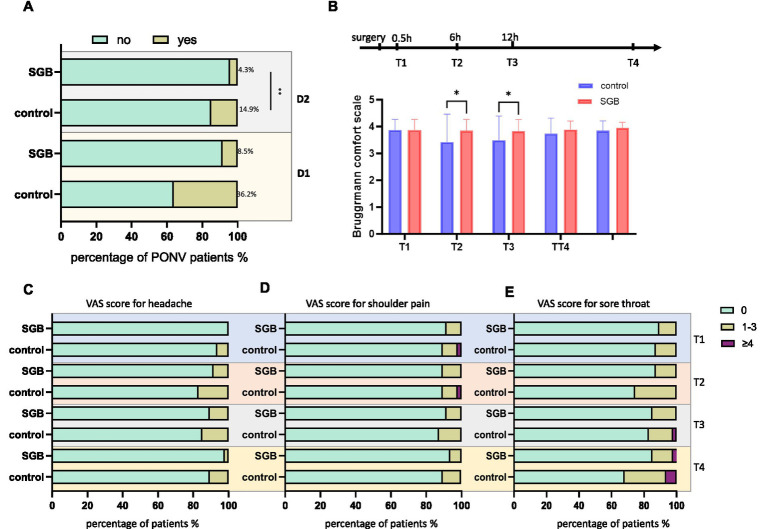
Comparison of postoperative nausea and vomiting, comfort, and different types of pain between the two groups. **(A)** Incidence of postoperative nausea and vomiting (PONV) on postoperative day 1 (D1) and day 2 (D2) in the two groups. Different colors indicate the presence or absence of PONV. **(B)** Brüggrmann comfort scale (BCS) scores at different postoperative time points in the two groups. T1, 30 min postoperatively; T2, 6 h postoperatively; T3, 12 h postoperatively; T4, 24 h postoperatively. **(C–E)** Distribution of visual analog scale (VAS) scores for postoperative headache, shoulder pain, and sore throat in the two groups. Different colors represent different pain intensity categories: 0, 1–3, and ≥4. Data are presented as mean ± standard deviation (SD) or percentage. *p < 0.05 vs. the control group.

Patients in the SGB group demonstrated a more favorable postoperative recovery profile than those in the control group. The incidence of postoperative nausea and vomiting (PONV) on postoperative day 1 was significantly lower in the SGB group (*p* < 0.05), while no significant difference was observed on postoperative day 2 (Table 4, Figure 4A). Early postoperative comfort, assessed using the Brüggrman Comfort Scale (BCS), was significantly higher in the SGB group at 30 min and 6 h after surgery (both *p* < 0.05). Differences at later time points (12 and 24 h) were not statistically significant (Table 6, Figure 4A). Postoperative pain outcomes, including headache, shoulder pain, and sore throat, as well as patient-controlled analgesia (PCA) usage, did not differ significantly between groups at any time point (all *p* > 0.05; Table 6, Figure 4C). Regarding functional recovery, the SGB group experienced an earlier time to first postoperative flatus and a significantly shorter length of hospital stay compared with the control group (both *p* < 0.05; Table 5, Figure 4D).

**Table 4:** Comparison of PONV between groups.

PONV (yes), *n*(%)	Control group (*n* = 47)	SGB group (*n* = 47)	Adjusted *p* value
D1	17 (36.2%)	4 (8.5%)	0.006*
D2	7 (14.9%)	2 (4.3%)	0.162

Values are presented as number (percentage). PONV, postoperative nausea and vomiting; SGB, stellate ganglion block. D0: preoperative; D1: postoperative day 1; D2: postoperative day 2. *p* values represent between-group comparisons and are adjusted for multiple comparisons. *indicates statistical significance.

**Table 5:** Comparison of perioperative hemodynamics, blood glucose, and postoperative recovery outcomes between groups.

Item	Control group (*n* = 47)	SGB group (*n* = 47)	*p* value
MAP (mmHg)			
t1	93 (14.5)	96 (15)	0.576
t2	75 (11.5)	77 (14.5)	0.181
t3	74 (9)	75 (12)	0.552
t4	94 (13)	87 (12)	0.066
HR (bpm)			
t1	76 (19.5)	75 (13.5)	0.652
t2	63 (10)	62 (9)	0.658
t3	62 (8.5)	60 (6)	0.991
t4	77 (15)	71 (12)	0.02*
GLU (mmol/L)			
t0	5.00 (1.15)	5.6 (1.25)	0.19
t1	6.20 (1.30)	6.10 (0.80)	0.118
t2	6.40 (1.30)	5.60 (1.27)	0.136
t3	5.00 (1.18)	5.60 (1.28)	0.109
Anal Exhaust time(h)	21 (6)	19 (7)	0.474
LOS(d)	9 (2)	8 (2)	0.037*

Data are presented as mean ± standard deviation (SD) or median (interquartile range, IQR). MAP, mean arterial pressure; HR, heart rate; LOS, length of stay. t0, pre-anesthesia baseline; t1, tracheal intubation; t2, skin incision; t3, 30 min after establishment of pneumoperitoneum; t4, tracheal extubation. *p < 0.05 vs. the control group.

**Table 6:** Comparison of postoperative comfort and pain-related outcomes between groups.

Item	Control group (*n* = 47)	SGB group (*n* = 47)	*p* value
BCS			
T1	3.43 ± 1.03	3.85 ± 0.41	0.028*
T2	3.49 ± 0.9	3.83 ± 0.43	0.038*
T3	3.74 ± 0.56	3.89 ± 0.31	0.214
T4	3.85 ± 0.36	3.95 ± 0.20	0.081
Postoperative pain			
Headache (yes), n(%)			
T1	5 (10.6%)	1 (2.1%)	0.092
T2	7 (14.9%)	7 (14.9%)	0.437
T3	8 (17.0%)	4 (8.5%)	0.201
T4	3 (6.4%)	0 (0.0%)	0.08
Shoulders pain (yes), n(%)			
T1	5 (10.6%)	3 (6.4%)	0.487
T2	6 (12.8%)	4 (8.5%)	0.507
T3	5 (10.6%)	5 (10.6%)	0.893
T4	5 (10.6%)	4 (8.5%)	0.651
Viscera pain (yes), n(%)			
T1	15 (31.9%)	7 (14.9%)	0.062
T2	8 (17.0%)	7 (14.9%)	0.808
T3	12 (25.5%)	6 (12.8%)	0.12
T4	6 (12.8%)	5 (10.6%)	0.65
PCA (times)	0 (1.5)	0 (0)	0.188

Data are presented as number (percentage) or mean ± standard deviation (SD). BCS, Brüggrmann Comfort Scale; VAS, visual analog scale; PCA, number of patient-controlled analgesia (PCA) bolus attempts. D1 and D2 indicate postoperative day 1 and postoperative day 2, respectively. T1, 30 min postoperatively; T2, 6 h postoperatively; T3, 12 h postoperatively; T4, 24 h postoperatively. *p < 0.05 vs. the control group.

#### Stress and autonomic markers

**Figure 5:**
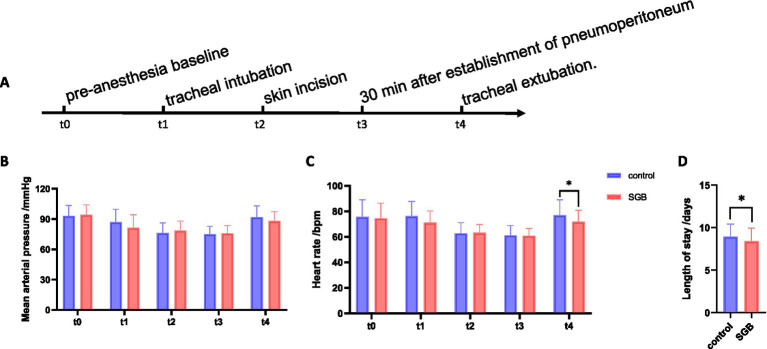
Comparison of perioperative hemodynamic changes and length of stay between the two groups. **(A)** Schematic illustration of perioperative time points. **(B)** Changes in heart rate (HR) at different perioperative time points in the two groups. **(C)** Changes in mean arterial pressure (MAP) at different perioperative time points in the two groups. **(D)** Comparison of postoperative length of stay (LOS) between the two groups. *p* < 0.05 vs. the control group.

No significant differences were observed between the two groups in heart rate (HR) or mean arterial pressure (MAP) before anesthesia induction, at skin incision, or 30 min after pneumoperitoneum establishment (all *p* > 0.05). However, at extubation, HR was significantly lower in the SGB group compared with the control group (*p* < 0.05), suggesting attenuation of perioperative sympathetic activation (Table 5; Figure 5B, C). Perioperative blood glucose levels did not differ significantly between groups at any measured time point (*p* > 0.05; Figure 5).

#### Safety outcomes

No serious adverse events related to stellate ganglion block were observed during the study period. A small proportion of patients in the SGB group developed transient Horner’s syndrome, which resolved spontaneously within several hours without requiring specific treatment.

## Discussion

This study observed that perioperative implementation of stellate ganglion block (SGB) was associated with improved early postoperative sleep quality in patients undergoing gynecological laparoscopic surgery. Compared with the control group, patients in the SGB group showed lower Athens Insomnia Scale (AIS) scores on postoperative days 1 and 2, as well as longer TST and a lower incidence of sleep disturbances. No significant differences in preoperative sleep status were observed between the two groups, which to some extent supports the notion that postoperative changes in sleep may be related to the SGB intervention. It has been reported that following gynecological laparoscopic surgery, the incidence of sleep disturbances is highest on postoperative days 1 and 2 (Song et al., 2025), which is consistent with the duration of action of SGB. Therefore, we evaluated early postoperative sleep quality on the first and second days after surgery, and the results suggest that SGB may have beneficial effects on both sleep quality and duration during this period. These findings indirectly support the concept that perioperative sleep is not merely a passive reflection of postoperative recovery but may represent a modifiable physiological process. From a clinical perspective, this suggests that modulation of autonomic nervous system function to improve sleep could serve as a potential perioperative management strategy, potentially exerting indirect benefits on overall recovery. Nevertheless, this hypothesis requires further investigation for confirmation.

Postoperative sleep disturbances are typically classified as acute sleep disorders, and their underlying mechanisms are believed to involve increased sympathetic activity, autonomic dysfunction, and dysregulation of the hypothalamic–pituitary–adrenal (HPA) axis (Fang et al., 2025). Previous studies have suggested that these changes may be closely related to perioperative stress responses. Various factors, including surgical trauma, anesthetic agents, postoperative pain, and psychological stress, can contribute to upregulation of sympathetic activity, thereby disrupting normal sleep–wake rhythms (Niklasson et al., 2025; Wang et al., 2025). In this context, autonomic dysfunction—particularly sustained sympathetic overactivity—is considered one of the potential physiological bases for reduced postoperative sleep quality. Given that stellate ganglion block (SGB) has been shown to modulate sympathetic activity, its beneficial effects on postoperative sleep may theoretically be related to autonomic regulation. However, this study did not directly assess autonomic function or related biomarkers, and therefore the above mechanisms should be regarded as speculative and require further mechanistic investigation for confirmation.

The stellate ganglion, as a key component of the cervical sympathetic chain, is involved in the regulation of cardiovascular responses, stress responses, and circadian rhythms (Jia et al., 2025; Sweeney, 1984). SGB, by transiently blocking sympathetic efferent activity, may influence perioperative autonomic function. First applied clinically in 1925, this technique has an overall favorable safety profile, with relatively few adverse effects, particularly when performed under ultrasound guidance (Lipov and Ritchie, 2015). In the present study, patients in the SGB group exhibited attenuated heart rate responses during high-stress time points, such as extubation, suggesting that sympathetic excitability may be partially suppressed. However, it should be noted that sympathetic activity was not directly measured in this study (e.g., via heart rate variability or skin conductance), and thus these findings can only serve as indirect indicators of autonomic modulation and should be interpreted with caution.

Furthermore, previous basic and clinical studies have suggested that sympathetic activity may play an important role in the initiation and maintenance of sleep (Jia et al., 2025; Campos et al., 2020; Cruz-Sanabria et al., 2024). The observed changes in heart rate and sleep parameters in this study—namely, reduced AIS scores and prolonged TST—allow us to hypothesize that SGB may exert effects on perioperative sleep through modulation of autonomic function. However, this study did not directly measure relevant neuroendocrine markers, such as melatonin or cortisol, and therefore this mechanism remains speculative and requires further investigation.

From a clinical perspective, SGB represents a short-term, controllable regional intervention that does not rely on central sedation. By influencing perioperative autonomic status, it may help modulate stress-related sleep disturbances, potentially reducing the need for pharmacological interventions and their associated adverse effects. In addition, the timing of SGB administration is well-defined, and its duration of action is relatively predictable, making it potentially valuable in the specific context of the perioperative period. Although SGB cannot replace conventional insomnia treatments, it may serve as an adjunctive strategy in the management of perioperative or stress-related sleep disturbances, providing a novel approach for individualized intervention.

Additionally, this study observed that patients in the SGB group had a lower incidence of postoperative nausea and vomiting, higher early comfort scores, and shorter hospital stays, changes that were directionally consistent with improvements in sleep. Previous studies have suggested that postoperative nausea and vomiting, decreased comfort, and delayed recovery may be associated with perioperative stress responses and autonomic dysfunction (Kehlet and Dahl, 2003). In the present study, patients in the SGB group experienced attenuation of these adverse outcomes alongside improvements in sleep parameters, suggesting a potential association between sleep quality and overall perioperative recovery. However, it should be noted that this study was not specifically designed to examine or infer causality between sleep improvement and these clinical outcomes; therefore, a causal relationship cannot be established. These findings are better interpreted as supplementary observations that support the primary results and provide directions for future research, rather than as direct evidence of causality.

This study has several limitations. First, postoperative sleep assessment primarily relied on subjective scales and wearable devices, without incorporating polysomnography. Second, as a single-center study with a relatively small sample size, the generalizability of the findings requires confirmation through multicenter, larger-scale studies. In addition, this study only evaluated postoperative sleep quality, and longer-term follow-up is planned for future research.

Furthermore, given that stellate ganglion block may induce transient Horner’s syndrome, an observer-blinded design was adopted, which could introduce some performance bias. Moreover, due to the small sample size, multiple secondary outcomes were analyzed—including headache, shoulder pain, sore throat, hemodynamic parameters, and length of hospital stay—and these results should be considered exploratory. As no systematic correction for multiple comparisons was performed, the risk of false-positive findings may be increased, and the statistical significance of these secondary outcomes should be interpreted with caution. Finally, autonomic function and neuroendocrine markers were not directly measured in this study, and the precise physiological mechanisms by which SGB improves sleep remain to be elucidated in future research.

## Conclusion

In summary, perioperative stellate ganglion block significantly improves early postoperative sleep in patients undergoing gynecological laparoscopic surgery. Sleep enhancement may represent an important component of the beneficial effects of SGB on overall perioperative recovery. These findings provide new clinical evidence supporting the potential role of SGB in perioperative management.

## Data Availability

The datasets presented in this study can be found in online repositories. The names of the repository/repositories and accession number(s) can be found in the article/Supplementary material.
