# Supplementary material for: Perioperative stellate ganglion block improves early postoperative sleep after gynecological laparoscopic surgery: a randomized controlled trial
Source: Front Neurosci. 2026 Apr 10;20:1820566. doi: 10.3389/fnins.2026.1820566 (PMC13106125; doi:10.3389/fnins.2026.1820566)
Supplement: Supplementary file 1 [file Supplementary_file_1.docx]

Table S1.

Comparison of postoperative sleep quality assessed by Athens Insomnia Scale (AIS) between the control group and SGB group

|  | control group (n=47) | SGB group (n=47) | *P* value |
| --- | --- | --- | --- |
| AIS D0 n(%) |  |  | 0.90 |
| ＜4 | 26(55.3%) | 25(53.2%) |  |
| 4-6 | 17(36.2%） | 20(42.5%) |  |
| ＞6 | 4(8.5%) | 2(4.3%) |  |
| AIS D1 n(%) |  |  | <0.001* |
| ＜4 | 10(21.3%) | 28(59.6%) |  |
| 4-6 | 28(59.6%) | 19(40.4%) |  |
| ＞6 | 9(19.1%） | 0（0%） |  |
| AIS D2 n(%) |  |  | 0.008* |
| ＜4 | 17（36.2%） | 30（63.8%） |  |
| 4-6 | 24(51.1%) | 17(36.2) |  |
| ＞6 | 6(12.8%) | 0(0%) |  |

alues are presented as number (percentage). AIS scores were categorized as follows: <4, no insomnia; 4–6, suspected insomnia; >6, insomnia. Comparisons between groups were performed using the chi-square test or Fisher’s exact test, as appropriate. A two-sided P value <0.05 was considered statistically significant.AIS, Athens Insomnia Scale; SGB, stellate ganglion block.

Table S2 Effect of SGB on postoperative AIS scores stratified by age

|  | <51 years SGB (n=12) | <51 years Control (n=14) | *P* value | ≥51 years SGB (n=32) | ≥51 years Control (n=33) | *P* value |
| --- | --- | --- | --- | --- | --- | --- |
| AIS (D0) | 3.67 ± 1.45 | 4.00 ± 1.36 | 0.528 | 3.59 ± 1.56 | 3.58 ± 1.82 | 0.966 |
| AIS (D1) | 3.33 ± 1.11 | 5.57 ± 1.40 | <0.001* | 3.41 ± 1.13 | 5.00 ± 1.98 | <0.001* |
| AIS (D2) | 3.33 ± 1.29 | 4.43 ± 0.94 | 0.014* | 3.28 ± 1.02 | 4.18 ± 1.86 | 0.019* |

Data are presented as mean ± standard deviation. Comparisons between groups were performed using independent-sample t-tests (Welch correction applied when appropriate). Interaction *P* values were calculated using a linear regression model including treatment group, age group (<51 vs. ≥51 years), and their interaction term. AIS: Athens Insomnia Scale; SGB: stellate ganglion block; D0: preoperativeD1: postoperative day 1; D2: postoperative day 2.

Table S3 Interaction analysis between age and SGB on postoperative AIS scores

|  | β (SGB) | β (Age group) | β (Interaction) | P for interaction |
| --- | --- | --- | --- | --- |
| AIS (D1) | -1.62 | -0.12 | 0.21 | 0.68 |
| AIS (D2) | -0.95 | -0.09 | 0.18 | 0.72 |

A linear regression model was used including treatment group (SGB vs. control), age group (<51 vs. ≥51 years), and their interaction term (SGB × age group). β (SGB) represents the overall treatment effect; β (Age group) represents the effect of age; β (Interaction) reflects whether the treatment effect differs between age groups.AIS: Athens Insomnia Scale; D1: postoperative day 1; D2: postoperative day 2.
